# Synthesis of Zirconium-Containing Polyhedral Oligometallasilsesquioxane as an Efficient Thermal Stabilizer for Silicone Rubber

**DOI:** 10.3390/polym10050520

**Published:** 2018-05-11

**Authors:** Jiedong Qiu, Xuejun Lai, Hongqiang Li, Xingrong Zeng, Zhuopeng Zhang

**Affiliations:** College of Materials Science and Engineering, Key Lab of Guangdong Province for High Property and Functional Polymer Materials, South China University of Technology, Guangzhou 510641, China; qiu.jiedong@mail.scut.edu.cn (J.Q.); hqli1979@gmail.com (H.L.); zhangzhuopeng01@126.com (Z.Z.)

**Keywords:** silicone rubber, zirconium-containing, polyhedral oligometallasilsesquioxane, thermal-oxidative stability, free-radical quenching

## Abstract

Free radicals play a negative role during the thermal degradation of silicone rubber (SR). Quenching free radicals is proposed to be an efficient way to improve the thermal-oxidative stability of SR. In this work, a novel zirconium-containing polyhedral oligometallasilsesquioxane (Zr-POSS) with free-radical quenching capability was synthesized and characterized. The incorporation of Zr-POSS effectively improved the thermal-oxidative stability of SR. The *T*_5_ (temperature at 5% weight loss) of SR/Zr-POSS significantly increased by 31.7 °C when compared to the unmodified SR. Notably, after aging 12 h at 280 °C, SR/Zr-POSS was still retaining about 65%, 60%, 75%, and 100% of the tensile strength, tear strength, elongation at break, and hardness before aging, respectively, while the mechanical properties of the unmodified SR were significantly decreased. The possible mechanism of Zr-POSS for improving the thermal-oxidative stability of SR was intensively studied and it was revealed that the POSS structure could act as a limiting point to suppress the random scission reaction of backbone. Furthermore, Zr could quench the free radicals by its empty orbital and transformation of valence states. Therefore, it effectively suppressed the thermal-oxidative degradation and crosslinking reaction of the side chains.

## 1. Introduction

Silicone rubber (SR) is widely used as encapsulation adhesive, sealant, and heat-insulating coating in the field of electronics and aerospace because of its excellent high-temperature resistance, ozone resistance, and electrical insulation [1,2,3]. SR maintains good service performance under 250 °C for a long time. However, with the rapid development of the high-power LED and aerospace industry, the working temperature of SR is getting higher and higher. When exposed above 280 °C in the air, the SR products suffer from the heat and oxygen attack. The backbone begins to degrade randomly and the side chains generate the methyl radical and the peroxyl radical, which results in the oxidative degradation and crosslinking. Consequently, the SR products become stiff and brittle and lose their mechanical properties and applied value [4,5,6]. To overcome this disadvantage, it’s necessary to prevent the random scission reaction of backbone and the oxidation of side chains. Many traditional thermal stabilizers such as metal oxide [7,8,9], graphene [10], carbon nanotube [11], and polyhedral oligomeric silsesquioxanes (POSS) [12,13,14] were introduced into the SR. Among these additives, POSS shows great potential for improving thermal stability of the polymer due to its unique intramolecular hybridization, designable structure, and good thermal stability. The nanoscale POSS could perform as a limiting point to suppress the thermal vibration of silicone chains and efficiently enhance the thermal-oxidative stability of SR [12]. It is noteworthy that the different organic moieties covalently bonded on silica core, which leads to diverse characteristics of POSS. For phenyl POSS, it possessed outstanding thermal stability and could form a ceramic protective barrier during thermal degradation to further improve the thermal-oxidative stability of SR [14].

As a derivative of POSS, polyhedral oligometallasilsesquioxane is a new kind of metal-containing organic-inorganic hybrid nanoparticle, which attracts more and more attention in recent years [15,16]. The introduction of metallic elements not only enhances the thermal stability of POSS, but also provides POSS with some advantageous metallic properties such as quenching free radicals by a change of valence states [7]. Moreover, the organic structure of POSS could effectively improve the compatibility between the metallic element and the polymer. Among many metallic elements, zirconium (Zr) has been considered to be one of the appropriate elements that could improve the thermal-oxidative stability of silicone resin [17,18]. Zr could receive the unpaired electrons by empty orbitals and change of valence states by the oxidation-reduction reaction, which indicates that Zr has a great potential in quenching free radicals [8,19]. However, the common Zr compounds such as zirconium oxide (ZrO_2_) were of great polarity and inhomogeneously dispersed in SR, which deteriorate the processability and mechanical properties of SR due to its poor compatibility with polymers. ZrO_2_ had relatively low efficiency. More than 10 wt % of ZrO_2_ was needed to endow SR with the satisfied thermal-oxidative stability [20]. Therefore, the main challenges encountered in developing desirable thermal stabilizers for SR were to improve the compatibility between the Zr compound and the polymer matrix and simultaneously improve its efficiency. 

Herein, we proposed an efficient method to overcome the issues by introducing Zr into POSS structure. In this work, zirconium-containing polyhedral oligometallasilsesquioxane (Zr-POSS) was synthesized via hydrolysis-condensation and corner capping reaction. Subsequently, it was introduced into SR to prepare the SR/Zr-POSS nanocomposite. It was found that Zr-POSS could effectively improve the thermal-oxidative stability of SR. The possible mechanism of Zr-POSS on the thermal-oxidative stability of SR was also revealed. This work provides a new way to prepare high-performance SR, which further promotes the development and application of SR.

## 2. Materials and Methods

### 2.1. Materials

Phenyltrimethoxysilane (PTMS) was supplied by Feidian Chem Co., Ltd., Hangzhou, China. NaOH and ZrCl_4_ were supplied by Shanghai Macklin Biochemical Technology Co., Ltd., Shanghai, China. Polymethylvinylsiloxane (PMVS, 110-8 and 110-7SK with vinyl content of 0.05 mol % and 2.93 mol %, and average molecular weight of 640,000 g/mol and 680,000 g/mol, respectively) gums were supplied by Shandong Dongyue Silicone Group Co., Ltd., Zibo, China. Polyhydromethylsiloxane (PHMS, Si-H: 1.2 wt %) was purchased from Square Silicone Co., Ltd., Shenzhen, China. 2,5-Bis(tert-butyl peroxide)-2,5-dimethyhexane (DBPMH, 50%) was supplied by Shinetsu Co., Ltd., Tokyo, Japan. Fumed silica (QS-20, 200 m^2^/g) was purchased from Tokuyama Chem Co., Ltd., Jiaxing, China. Toluene, tetrahydrofuran (THF), isopropanol, and methanol were purchased from Bodi Chemical, Tianjin, China. All the materials were used directly without further purification.

### 2.2. Synthesis of Zr-POSS

First, Na_4_O_14_Si_8_(C_6_H_5_)_8_ was synthesized according to the reference [21]. Exactly 96.0 g (0.48 mol) PTMS, 10.0 g (0.55 mol) H_2_O, 12.8 g (0.32 mol) NaOH, and 480 mL isopropanol were added in a 1 L round bottom flask. The mixture was heated and kept under reflux for 4 h. Subsequently, the system was cooled to room temperature and the reaction was conducted for another 24 h. Afterward, the solvent was removed by filtration. The residue was washed with isopropanol three times and dried in a vacuum oven at 40 °C for 24 h to obtain Na_4_O_14_Si_8_(C_6_H_5_)_8_ (48.0 g, 68.3%).

**Figure 1:**
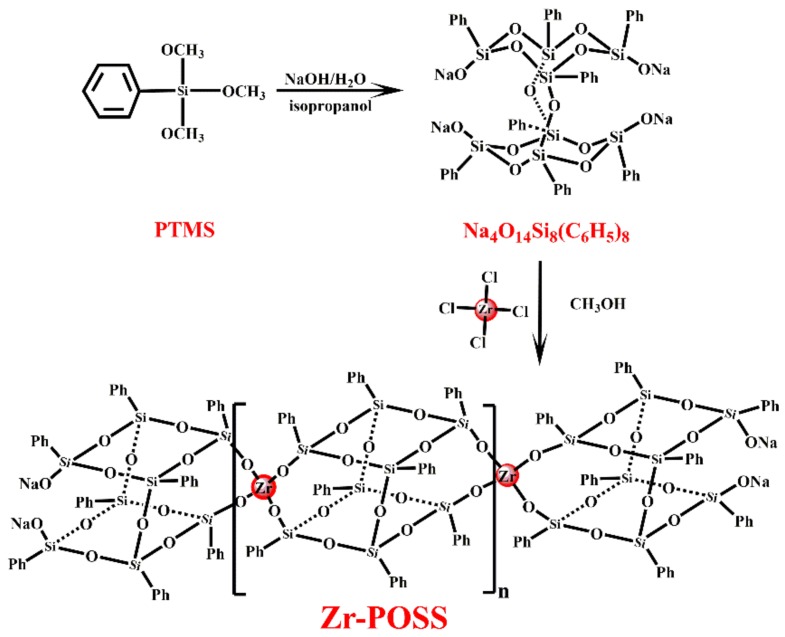
Synthetic route of Zr-POSS.

Next, 11.54 g (0.01 mol) Na_4_O_14_Si_8_(C_6_H_5_)_8_, 2.56 g (0.01 mol) ZrCl_4_, and 200 mL methanol were added in a 500 mL round bottom flask. Afterward, the mixture was stirred at 65 °C for 24 h under nitrogen. Next, the methanol was removed under the reduced pressure and the solid was re-dissolved with THF. The mixture was filtered and THF was removed under the reduced pressure to obtain Zr-POSS (11.0 g, 95.2%). The synthetic route of Zr-POSS was shown in Figure 1.

### 2.3. Preparation of SR/Zr-POSS Nanocomposites

Furthermore, 10 g of Zr-POSS, 30 g of SiO_2_, and 500 mL toluene were added in a 1000 mL round bottom flask. Then the mixture was stirred at room temperature for 1 h. Afterwards, the toluene was removed to obtain the Zr-POSS/SiO_2_ mixture. 100 g of PMVS gum (the mass ratio of 110-8 and 110-7SK was 93:7), 0.6 g of PHMS, 2 g of DBPMH, 0~16 g of Zr-POSS/SiO_2_ mixture and 3~15 g of SiO_2_ (the total amount of SiO_2_ in system was maintained in 15 g) were mixed uniformly by a two-roll mill to obtain a transparent compound. After that, the mixture was vulcanized at 155 °C for 15 min (according to the results of vulcanization test (see Supplementary Materials Table S1)) under 8 MPa to obtain the SR/Zr-POSS nanocomposites. Different nanocomposites with the addition of Zr-POSS including 1, 2, 3, and 4 phr (per hundreds of rubber) were obtained in the system. The samples were named in the form of SR/Zr-POSS-*x*. “*x*” stood for the content of Zr-POSS to SR.

### 2.4. Characterization

#### 2.4.1. Fourier Transform Infrared Spectrometry (FTIR)

FTIR spectra of the samples were examined by a Tensor 27 spectrometer (Bruker Optics, Karlsruhe, Germany). The wavenumber range was 4000–400 cm^−^^1^ and the resolution was 4 cm^−^^1^.

#### 2.4.2. ^29^Si Nuclear Magnetic Resonance Spectrometry (^29^Si-NMR)

The ^29^Si-NMR spectrum of the sample was conducted by an AVANCE AV-400 Fourier transform superconducting magnetic resonance spectrometer (Bruker, Karlsruhe, Germany).

#### 2.4.3. Gel Permeation Chromatography (GPC)

GPC was performed with a Waters 515 HPLC pump (Waters) equipped with a Shodex K-G guard column and a Shodex K-804L chromatographic column. Detection was achieved with a Waters 2414 refraction index detector and the samples were analyzed at 30 °C with chloroform as the eluent at a flow rate of 1 mL/min. The instrument was calibrated with low poly dispersed polystyrene standards.

#### 2.4.4. Transmission Electron Microscopy (TEM)

The morphology of Zr-POSS and its nanocomposite was observed by transmission electron microscopy (JEM-2100F, Tokyo, Japan). The nanocomposite was cryo-cut with a diamond knife into a thickness of 50 nm to 70 nm.

#### 2.4.5. Scanning Electron Microscope (SEM)

The morphology of the Zr-POSS and its nanocomposite was observed by using SEM (EVO18, Carl Zeiss Jena, Co, Oberkochen, Germany) with energy-dispersive X-ray spectroscopy (EDS).

#### 2.4.6. Thermogravimetry Analysis (TGA)

TGA was determined by a thermo-gravimeter (TG209, Netzsch Instruments, Selb, Germany) from 25 °C to 800 °C with a heating rate of 20 °C·min^−1^ under air or nitrogen atmosphere.

#### 2.4.7. Thermogravimetry-Fourier Transform Infrared Spectrometry (TG-FTIR)

The TG-FTIR was studied using a thermo-gravimeter instrument (TG209, Netzsch Instruments, Selb, Germany) with a Fourier transform infrared spectrometer (Tensor 27, Bruker Optics, Selb, Germany) from 25 °C to 800 °C with a heating rate of 20 °C·min^−1^ under air or nitrogen atmosphere.

#### 2.4.8. X-ray Photoelectron Spectroscopy (XPS)

XPS was recorded on an X-ray photoelectron spectrometer (Kratos Axis Ulra DLD, Shimadzu, Japan) by employing a monochromatic Al Kα X-ray source. The range of the binding energy was 174.9 eV to 188.0 eV.

#### 2.4.9. Mechanical Tests

The tensile strength and tear strength of SR samples were measured by using a tensile tester (UT-2060, U-CAN Dynatex Inc., Nantou, Taiwan) with a crosshead speed of 500 mm·min^−1^, according to ASTM A370-03a (American Society for Testing and Materials, Philadelphia, PA, USA) and ASTM 624-00 (American Society for Testing and Materials, Philadelphia, PA, USA).

#### 2.4.10. Cross-link Density Test

The crosslinking density of vulcanizates (Ve) and average molecular weight between crosslinking knots (Mc) were evaluated via the swelling equilibrium experiment as a reference [11].

Ve = M/Mc(1)

A certain amount of samples was immersed in 50 mL toluene for 72 h at 25 °C to attain equilibrium swelling. After the equilibrium swelling, the sample was taken out and the solvent was blotted from the surface of the sample and weighted immediately. Mc was calculated by the Flory-French Equation (1).

(2) $$\mathrm{Mc}=\frac{-\rho_{2}V_{0}\varphi^{\frac{1}{3}}}{\ln\left(1-\varphi \right)\;+\;\varphi \;+{\;\chi \varphi }^{2}}$$

where *V*_0_ is the molar volume of the solvent, χ is Flory-Huggins interaction parameter between polymer and solvent, here is 0.465 [11] and φ is the volume fraction of SR in the swelling rubber, which can be obtained through Equation (2).

(3) $$\Phi =\frac{W_{1}/\rho_{2}}{(W_{2}-W_{1})/\rho_{1}+W_{1}/\rho_{2}}$$

where ρ_1_ and ρ_2_ are the densities of toluene and SR, respectively. *W*_1_ is the initial weight of SR composite and *W*_2_ is the weight of swollen SR composite.

#### 2.4.11. Thermal-Oxidative Aging of the SR/Zr-POSS Nanocomposites

The vulcanizates were aged in an air-blowing oven at 280 °C for 12 h.

## 3. Results and Discussions

### 3.1. Characterization of Zr-POSS

**Figure 2:**
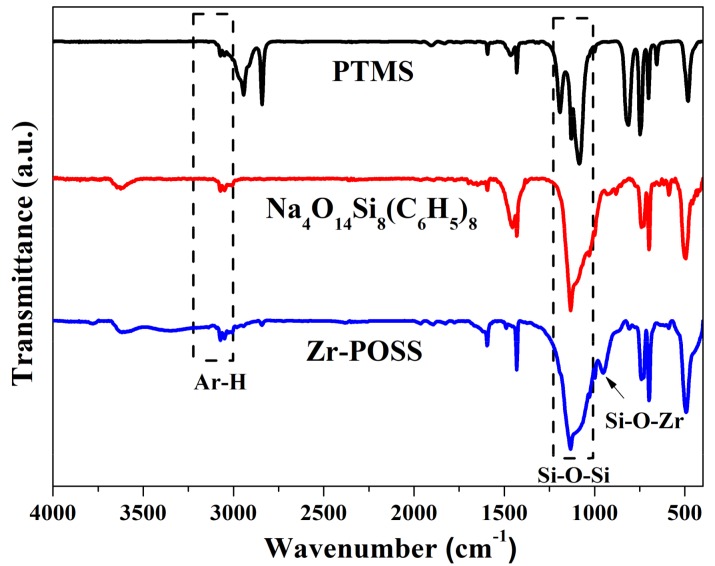
FTIR spectra of PTMS, Na_4_O_14_Si_8_(C_6_H_5_)_8_ and Zr-POSS.

**Figure 3:**
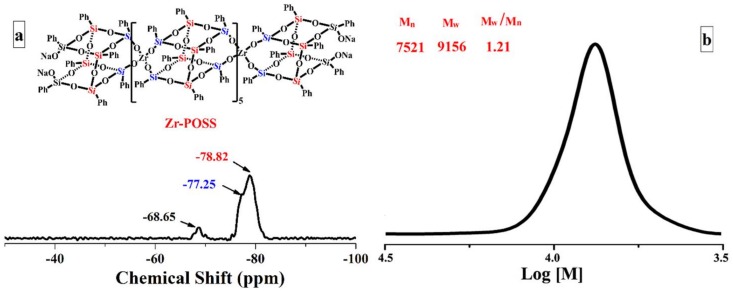
^29^Si-NMR (**a**) and GPC elution (**b**) curve of Zr-POSS.

Figure 2 shows the FTIR spectra of PTMS, Na_4_O_14_Si_8_(C_6_H_5_)_8_, and Zr-POSS. As illustrated in Figure 2, PTMS was mainly characterized by the absorptions of 3073–3051 cm^−1^ (ν_Ar–H_), 2942–2844 cm^−1^ (ν_C–H_), 1593 cm^−^^1^ (ν_Ar-C=C_), and 1084 cm^−1^ (ν_Si‒O‒C_) [22]. After the hydrolysis-condensation of PTMS, the ν_C–H_ peak belonging to Si–O–CH_3_ disappeared completely and a strong peak appeared at 1132 cm^−1^, which was attributed to the Si–O–Si stretching vibration of core in Na_4_O_14_Si_8_(C_6_H_5_)_8_ [21]. When Na_4_O_14_Si_8_(C_6_H_5_)_8_ reacted with ZrCl_4_, a new absorption band ascribed to the Si–O–Zr appeared at 954 cm^−1^ [23], which meant that Zr-POSS was synthesized. Figure 3 shows the ^29^Si-NMR and GPC elution curve of Zr-POSS. The ^29^Si NMR spectrum of Zr-POSS (see Figure 2a) had three signals at −78.82 ppm, −77.25 ppm, and −68.65 ppm. The characteristic signals located at −77.25 ppm and −78.82 ppm correspond to Ph-Si-(OSi)_2_-(OZr) and Ph-Si-(OSi)_3_, respectively [24]. The peak at −68.65 ppm could be attributed to Ph-Si-(OSi)_2_-(ONa) in the terminal of chain. Deconvolution analysis is an appropriate method to ascertain the polymerization degree of coordination polymers [15]. After the deconvolution of ^29^Si NMR spectrum, the integration ratio of Ph-Si-(OSi)_2_-(ONa) to Ph-Si-(OSi)_2_-(OZr) and Ph-Si-(OSi)_3_ type silicon signal was approximately 1:13. Based on the molecular formula of Zr-POSS, the number of Ph-Si-(OSi)_2_-(ONa), Ph-Si-(OSi)_2_-(OZr), and Ph-Si-(OSi)_3_ were 4, 4 × (n + 1), and 4 × (n + 2), respectively (n was the degree of polymerization for Zr-POSS). On the basis of these results, the degree of polymerization for Zr-POSS was 5. In Figure 3b, the Mn and Mw of Zr-POSS were 7521 g/mol and 9156 g/mol, respectively. This result indicated that the degree of polymerization for Zr-POSS was about 5, which was accordant with the result of ^29^Si-NMR spectra.

**Figure 4:**
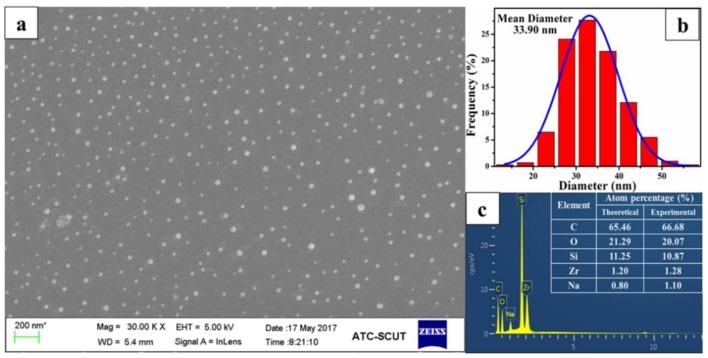
SEM (**a**); diameter distribution (**b**); and SEM-EDS (**c**) of Zr-POSS.

Figure 4 shows the SEM, diameter distribution, and SEM-EDS of Zr-POSS. As displayed, Zr-POSS was a spherical particle with a mean diameter of 33.9 nm. The atom percentage of C, O, Na, Si, and Zr was 66.68%, 20.07%, 1.10%, 10.87%, and 1.28%, which were approximately in accordance with their theoretical percentage, respectively. Based on the above analysis, it was further confirmed that Zr-POSS had been synthesized successfully.

### 3.2. Morphology of the SR/Zr-POSS Nanocomposites

**Figure 5:**
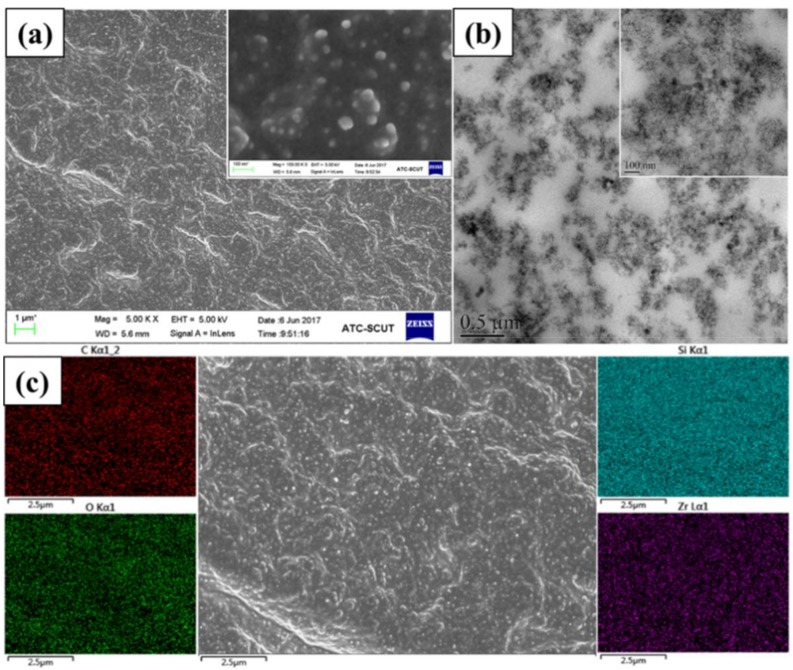
SEM (**a**); TEM (**b**); and SEM-EDS mapping (**c**) images of SR/Zr-POSS-4 nanocomposite.

The distribution of nanofiller in the matrix is of great importance for the properties of nanocomposites. Figure 5 shows SEM, TEM, and SEM-EDS mapping digital images of SR/Zr-POSS nanocomposites. The continuous phase was silicone rubber and the dispersed phase was Zr-POSS. When Zr-POSS was added into SR, the particle size of Zr-POSS still remained in about 34 nm and no aggregate was found in SEM and TEM. The Zr element dispersed homogeneously in SEM-EDS mapping (see Figure 5c), which indicated that Zr-POSS was uniformly distributed in SR.

### 3.3. Thermal-Oxidative Aging Properties of the SR/Zr-POSS Nanocomposites

**Figure 6:**
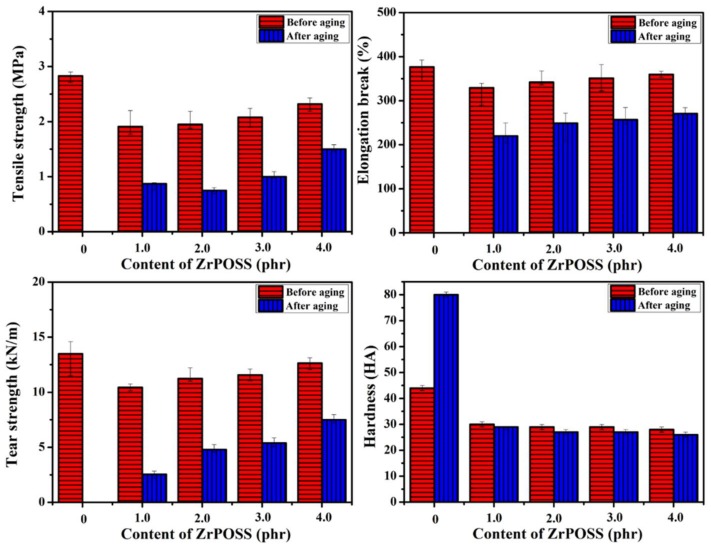
Mechanical properties of the SR/Zr-POSS nanocomposites before and after thermal-oxidative aging.

The mechanical properties for SR/Zr-POSS nanocomposites before and after thermal-oxidative aging are presented in Figure 6. The unmodified SR after aging showed a sharp rise in hardness and became brittle and even dehiscent, which was attributed to the oxidative degradation and cross-link of the side methyl groups. As a result, the tensile strength and tear strength of the treated unmodified SR were too weak to be measured. When Zr-POSS was added, the mechanical properties of the SR/Zr-POSS nanocomposites after thermal-oxidative aging were remarkably improved. For the treated SR/Zr-POSS-4, the tensile strength decreased from 2.3 MPa to 1.5 MPa while the elongation at break decreased from 360.0% to 271.0% and the tear strength decreased from 12.6 kN/m to 7.5 kN/m, respectively. Notably, the hardness remained almost the same as before aging. These phenomena suggested that Zr-POSS could greatly improve the thermal-oxidative aging property of SR. On one hand, the POSS of Zr-POSS effectively enhanced the thermal stability of SR by its large steric effect, excellent thermal stability, and an intense coupling effect with main chains of SR [14]. On the other hand, during the aging process, Zr^4+^ could not only receive the unpaired electrons of the free radical by its empty orbital but also transfers the free radical into stable compounds with the effect of valence change [8,19].

### 3.4. Thermal-Oxidative Aging Mechanism of the SR/Zr-POSS Nanocomposites

#### 3.4.1. Thermal-Oxidative Stability

**Figure 7:**
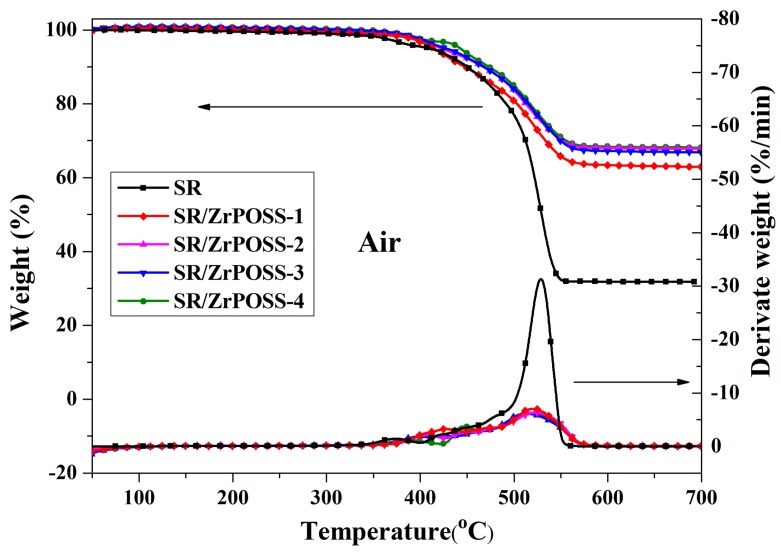
TGA and DTG curves of SR/Zr-POSS nanocomposites under air atmosphere.

Figure 7 presents the TGA and DTG curves of SR/Zr-POSS nanocomposites under air atmosphere and the characteristic results are listed in Table 1. The *T*_5_ (temperature at 5% weight loss) of SR was 411.5 °C, which was mainly attributed to the oxidation degradation of the side group of methyl [25]. With the increase of temperature, SR decomposed rapidly and reached the *T*_1max_ (temperature at maximum weight loss rate) at 453.3 °C, which was ascribed to the random scission reaction of the SR backbones [26]. When combined with 1.0 phr Zr-POSS, the *T*_5_ of SR/Zr-POSS-1 nanocomposite was 415.3 °C, and the *T*_1max_ was increased from 369.0 °C to 427.1 °C while the maximum degradation rate (*R*_max_) was decreased from 32.5 wt %/min to 7.00 wt %/min, which means that a small amount of Zr-POSS could remarkably improve the thermal-oxidative stability of SR. When the Zr-POSS content was increased, the thermal-oxidative stability of SR was further improved. 4.0 phr Zr-POSS could increase *T*_5_ and the residue at 700 °C of SR increased by 31.7 °C and 36.4 wt %, respectively. These might be due to the rigidity of the benzene ring and the inorganic silica core, which could improve the thermal stability of SR by limiting the movement of the molecular chain. Moreover, the empty orbital of transition metal Zr^4+^ could receive the unpaired electrons of the free-radical produced by the oxidation and degradation of SR. During the thermal aging, Zr^4+^ was partly converted into Zr^3+^ or Zr^2+^ by capturing free radicals. Additionally, the phenyl of Zr-POSS with a larger conjugate system could react with free radicals to form coordination bonds, which effectively passivated the free radical [27,28].

#### 3.4.2. Thermal-Oxidative Stability

To further investigate the effect of Zr-POSS on the thermal-oxidative stability of SR, the average molecular weight between crosslinking knots (Mc) of the SR/Zr-POSS nanocomposites before and after aging was characterized and the results are shown in Table 2. Smaller Mc means higher crosslinking density [9]. During the vulcanization of SR, the peroxide vulcanizing agent produces free radicals to initiate the cross-linking reaction of SR. When Zr-POSS was incorporated into SR, it would trap some free radicals by the valence change and have some negative effect on the vulcanization of SR. As a result, the Mc of SR increased by adding Zr-POSS. However, the SR/Zr-POSS nanocomposites could be cured normally and the effect of Zr-POSS on the curing for SR was negligible (see Table S1). In addition, when the SR/Zr-POSS nanocomposites were placed in the air, the low valence states of Zr (+3 and +2) were oxidized into a high and stable valence state (+4)to guarantee the persistence of free-radical quenching. After aging, the Mc of the unmodified SR decreased from 4338 to 1526, which reduced by 64.8%. It confirmed that the oxidation and fracture of the side methyl group led to a further crosslink reaction of SR during the thermal-oxidative aging. By contrast, the Mc of the SR/Zr-POSS nanocomposites had no clear change after aging. Especially when the content of the Zr-POSS was 4.0 phr, the Mc of SR/Zr-POSS-4 nanocomposites was almost unchanged, which further confirmed that Zr-POSS played an important role in improving the thermal-oxidative stability of SR.

#### 3.4.3. Attenuated Total Reflection (ATR)

**Figure 8:**
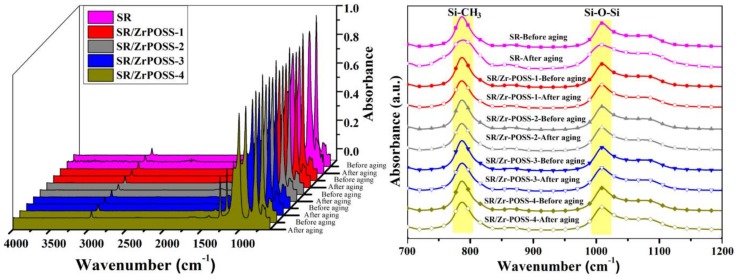
ATR spectra of the SR/Zr-POSS nanocomposites before and after aging.

In order to further explore the influence of Zr-POSS on the thermal-oxidative stability of SR, it is necessary to study the changing status of the side methyl groups. Figure 8 shows the ATR spectra of SR/Zr-POSS nanocomposites before and after thermal-oxidative aging and the characteristic data are listed in Table 3. There are two major bands belonging to Si-O-Si (1008 cm^−1^) and Si-CH_3_ (788 cm^−1^) in the ATR spectra [29]. Since the thermal aging of SR was mainly the oxidation of Si-CH_3_ under air atmosphere, the conservation rate (R) of the side methyl group calculated by the following formula could be regarded as the thermal-oxidative stability of SR/Zr-POSS nanocomposites.

(4) $$R=\frac{{\mathrm{Abs}}_{\mathrm{Si}-\mathrm{CH}3}\left(\mathrm{after}\;\mathrm{aging}\right)\times {\mathrm{Abs}}_{\mathrm{Si}-O-\mathrm{Si}}\left(\mathrm{before}\;\mathrm{aging}\right)}{{\mathrm{Abs}}_{\mathrm{Si}-O-\mathrm{Si}}\left(\mathrm{after}\;\mathrm{aging}\right)\times {\mathrm{Abs}}_{\mathrm{Si}-\mathrm{CH}3}\left(\mathrm{before}\;\mathrm{aging}\right)}$$

As previously seen, after aging at 280 °C for 12 h under air atmosphere, the R value of the unmodified SR was only 69%, which demonstrates that the side methyl group was destructed by the heat and oxygen. When Zr-POSS was incorporated into SR, the *R* value of SR/Zr-POSS nanocomposites was improved. As for SR/Zr-POSS-4, the *R* value was up to 95%, which indicated that the addition of Zr-POSS could effectively prevent the side methyl group from degradation.

#### 3.4.4. XPS

**Figure 9:**
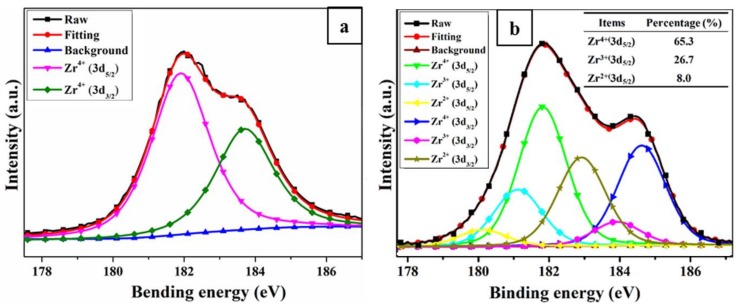
XPS spectrum of Zr(3d) for SR/Zr-POSS-4 nanocomposites before (**a**) and after aging (**b**).

In order to further confirm that Zr-POSS could trap free radicals by the effect of valence change during the thermal-oxidative aging of SR, XPS were used to analyze the valence of Zr. Figure 9 shows the XPS spectrum of Zr(3d) for SR/Zr-POSS-4 before and after thermal-oxidative aging. The observed Zr 3d_3/2_ and Zr 3d_5/2_ at 183.7 eV and 181.9 eV (in Figure 9a) indicate that zirconium was in the +4 oxidation state. After aging, the Zr(3d) XPS spectrum of SR/Zr-POSS-4 retained the peaks of Zr^4+^. Interestingly, the peaks assigned to Zr^3+^ (3d_5/2_ = 181.2 eV, 3d_3/2_ = 183.0 eV) and Zr^2+^ (3d_5/2_ = 180.2 eV, 3d_3/2_ = 182.0 eV) appeared in the system and the percentage of peak area for Zr^4+^, Zr^3+^, and Zr^2+^ were 65.3%, 26.7%, and 8.0%, respectively [30]. The results indicated that during the thermal-oxidative aging, Zr^4+^ would partly convert into Zr^3+^ and Zr^2+^ by capturing free radicals. After that, under the action of oxygen, the low valence states of Zr (+3 and +2) were oxidized into high and stable valence states (+4). Consequently, a reduction-oxidation cycle was formed to guarantee the persistence of free radical quenching and, therefore, effectively improved the thermal-oxidative stability of SR.

#### 3.4.5. TG-FTIR

**Figure 10:**
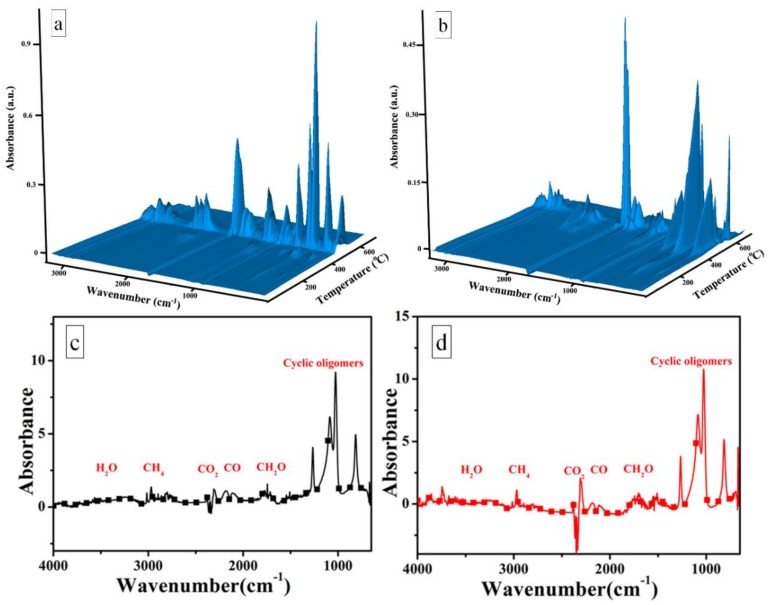
3D TG-FTIR of thermal degradation products for SR (**a**) and SR/Zr-POSS-4 nanocomposites (**b**) and FTIR spectra of thermal degradation products for SR (**c**) and SR/Zr-POSS-4 nanocomposites (**d**) under air atmosphere.

TG-FTIR was also used to analyze the evolution of the pyrolysis products during the thermal degradation. Figure 10 shows the 3D TG-FTIR and FTIR spectra of pyrolysis products for SR (a) and SR/Zr-POSS-4 nanocomposites (b) under air atmosphere. The main gaseous decomposition products of the unmodified SR and the SR/Zr-POSS nanocomposites were recognized explicitly by the characteristic signals of FTIR such as cyclic oligomers (849 cm^−1^ and 1026 cm^−1^), CH_2_O (1745 cm^−1^), CO (2179 cm^−1^ and 2114 cm^−1^), CO_2_ (2359 cm^−1^ and 2314 cm^−1^), methane (3017 cm^−1^ and 1304 cm^−1^), and H_2_O (3500–3700 cm^−1^) [31].

**Figure 11:**
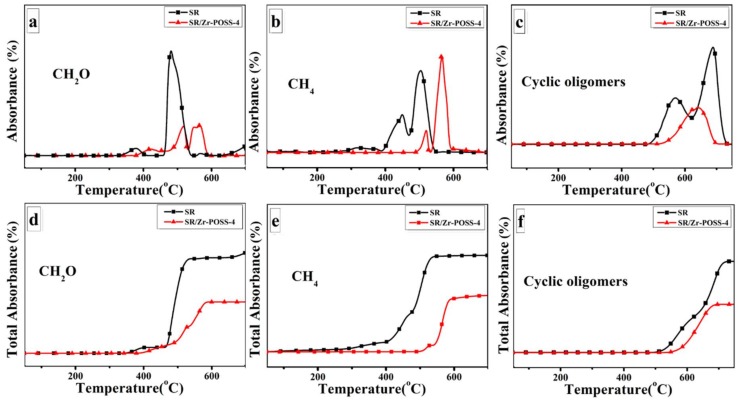
FTIR absorbance evolution of CH_2_O (**a**), CH_4_ (**b**) and cyclic oligomers (**c**), and total absorbance of CH_2_O (**d**), CH_4_ (**e**) and cyclic oligomers (**f**) during thermal degradation for SR and SR/Zr-POSS-4 under air atmosphere.

In order to reveal the degradation pathway of unmodified SR and SR/Zr-POSS nanocomposites, the absorbance of several representative thermo-oxidative degradation products for polymer versus temperature was analyzed and the results were illustrated in Figure 11. The unmodified SR began to degrade at 320 °C to produce CH_2_O and reached the first and second peaks at about 383 °C and 481 °C, respectively. Under high temperature, oxygen could oxidize the methyl group of SR into peroxides, which were unstable and easily broke down into CH_2_O [26]. When the temperature reached up to 398 °C, the methyl radical generated by the breakage of Si-C bonds would react with the nearby methyl to release methane and reach the peaks at 443 °C and 503 °C [32]. Synchronously, the molecular chains with free radicals reacted with each other to form Si–C–Si bonds, which lead to the further cross-link of SR [33]. In addition, with the increase of temperature, the cyclic oligomers were generated by the unzipping reaction of Si-OH end-groups and the random scission reaction of backbone.

When 4.0 phr Zr-POSS was incorporated into SR, the pyrolysis products of SR/Zr-POSS-4 showed some differences from those of the unmodified SR. As shown in Figure 11a, the incorporation of Zr-POSS delayed the first and the second release peaks of CH_2_O by 37 °C and 36 °C, respectively. In addition, the total amount of CH_2_O was also suppressed. For methane and cyclic oligomers, the temperature of the first release peaks increased to 521 °C and 624 °C, which were 78 °C and 57 °C higher than those of the unmodified SR, respectively. Therefore, Zr-POSS could delay the production of CH_2_O, methane, and cyclic oligomers. This may be because Zr-POSS could suppress the random scission reaction of backbone and timely quench the methyl radical and peroxide radical to protect the matrix from degradation.

#### 3.4.6. Possible Mechanism

**Figure 12:**
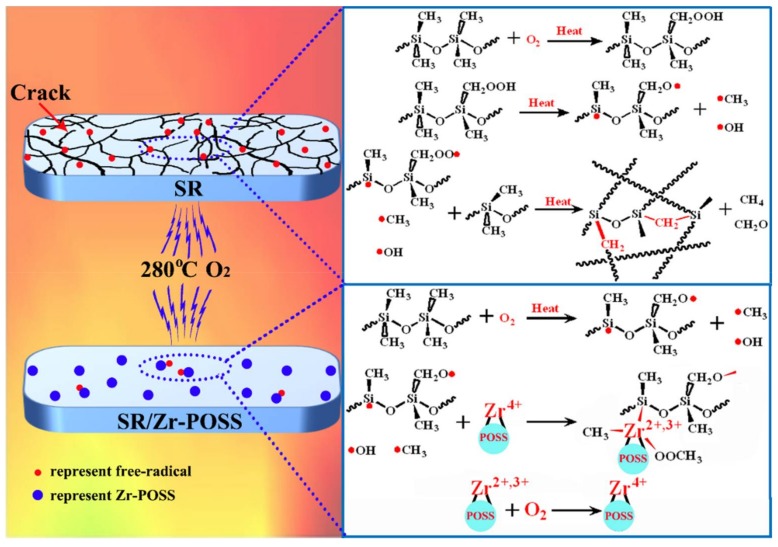
Possible mechanism of Zr-POSS on improving the thermal-oxidative aging properties of SR.

Based on the analysis mentioned above, the possible mechanism of Zr-POSS for SR was proposed and shown in Figure 12. When being attacked by the high-temperature oxygen above 280 °C, plenty of methyl radicals and peroxyl radicals were produced in SR, which led to the further degradation and crosslinking of the side chains. Accordingly, the hardness of SR increased sharply and the mechanical properties were lost completely.

For SR/Zr-POSS, Zr-POSS showed a positive effect on the thermal-oxidative stability of SR. Because of the POSS, Zr-POSS could perform as a limiting point to suppress the random scission reaction of backbone and effectively enhance the thermal stability of SR. When being attacked by the heat and oxygen, Zr-POSS played its role in quenching the methyl radical and peroxyl radical, which were generated by SR degradation. On one hand, the empty orbital of Zr^4+^ could receive the unpaired electrons of free radicals to limit its activity. On the other hand, Zr^4+^ could transfer the free radical into stable compounds and converted into Zr^3+^ and Zr^2+^. Interestingly, Zr^3+^ or Zr^2+^ could be oxidized into Zr^4+^ again by forming a reduction-oxidation cycle to ensure the continuity of the free-radical quenching. Therefore, Zr-POSS effectively improved the thermal-oxidative stability of SR.

## 4. Conclusions

A novel zirconium-containing polyhedral oligometallasilsesquioxane (Zr-POSS) was successfully synthesized and characterized. Zr-POSS could effectively improve the thermal stability of SR. When the content of Zr-POSS was 4.0 phr, the *T*_5_ under air atmosphere was increased by 31.7 °C. More importantly, Zr-POSS could remarkably improve the thermal-oxidative aging properties of SR. After aging for 12 h at 280 °C, SR/Zr-POSS was still retaining most of its tensile strength, tear strength, elongation at break, and hardness. The mechanism revealed that when the Si-CH_3_ groups of SR were attacked by hot oxygen and degraded into free-radicals, Zr-POSS could effectively trap the free-radicals and transfer them into stable compounds. Zr-POSS played an important role in quenching free radicals and suppressing the oxidative degradation of SR. This work provided a new way to prepare high temperature resistant polymers.

## Figures and Tables

**Table 1:** Characteristic data obtained from TGA and DTG curves (air, 20 °C/min).

Samples	Zr-POSS Content (^a^ phr)	*T*_5_ (°C)	*T*_1max_ (°C)	*T*_max_ (°C)	*R*_max_ (wt %/min)
SR	0	411.5	369.0	529.1	32.5
SR/Zr-POSS-1	1	415.3	427.1	520.1	7.00
SR/Zr-POSS-2	2	423.6	398.5	512.7	6.14
SR/Zr-POSS-3	3	428.1	406.2	522.8	6.32
SR/Zr-POSS-4	4	443.2	386.7	518.5	6.34

^a^ Parts per hundred of rubber.

**Table 2:** Mc of the SR/Zr-POSS nanocomposites before and after thermal-oxidative aging.

Samples	^1^ Mc		Decreasing Amplitude (%)
	Before Aging	After Aging	
SR	4338 ± 163	1526 ± 134	64.8
SR/Zr-POSS-1	6985 ± 123	5668 ± 147	18.8
SR/Zr-POSS-2	7597 ± 138	6669 ± 129	12.2
SR/Zr-POSS-3	7616 ± 148	7208 ± 152	5.4
SR/Zr-POSS-4	6845 ± 126	6827 ± 135	0.3

^1^ Mc = average molecular weight between crosslinking knots.

**Table 3:** Characteristic data obtained from ATR.

Samples	Aging	Abs_Si–O–Si_ (1008 cm^−^^1^)	Abs_Si-CH3_ (788 cm^−^^1^)	Conservation Rate (%)
SR	Before	0.760	0.957	69.0
	After	0.763	0.663	
SR/Zr-POSS-1	Before	0.738	0.930	91.2
	After	0.773	0.890	
SR/Zr-POSS-2	Before	0.734	0.937	92.4
	After	0.763	0.900	
SR/Zr-POSS-3	Before	0.732	0.936	93.5
	After	0.638	0.763	
SR/Zr-POSS-4	Before	0.714	0.910	95.0
	After	0.723	0.875	

## Supplementary Materials

The following are available online at http://www.mdpi.com/2073-4360/10/5/520/s1, Table S1: Effect of Zr-POSS on the curing characteristics of SR, Table S2: Characteristic data obtained from TGA and DTG curves (nitrogen, 20 °C/min), Figure S1: UV-Vis spectra of SR/Zr-POSS nanocomposites, Figure S2: TGA and DTG curves of SR/Zr-POSS nanocomposites under nitrogen atmosphere, Figure S3: 3D TG-FTIR and FTIR spectra of pyrolysis products for SR (a) and SR/Zr-POSS-4 nanocomposites (b) under nitrogen atmosphere, Figure S4: FTIR absorbance evolution of the pyrolysis products during thermal degradation for SR and SR/Zr-POSS-4 nanocomposites under nitrogen atmosphere, Figure S5: ^29^Si NMR spectra of SR (a) and SR/Zr-POSS-4 nanocomposites (b) before and after aging.
