# Supplementary material for: Synthesis of Zirconium-Containing Polyhedral Oligometallasilsesquioxane as an Efficient Thermal Stabilizer for Silicone Rubber
Source: Polymers (Basel). 2018 May 11;10(5):520. doi: 10.3390/polym10050520 (PMC6415425; doi:10.3390/polym10050520)
Supplement: Supplementary file 1 [file polymers-10-00520-s001.pdf]

polymers MDPI

S1. Curing of SR/Zr-POSS nanocomposites

Table S1. Effect of Zr-POSS on the curing characteristics of SR.

Zr-POSS (phr)	T_{10} (s)	T_{90} (s)	M_L (dN·m)	M_H (dN·m)	ΔM (dN·m)
0	85	481	2.29	11.90	9.71
1.0	85	474	1.08	6.53	5.45
2.0	90	485	0.87	6.11	5.24
3.0	98	574	0.87	6.40	5.53
4.0	103	583	0.78	6.19	5.41

Table S1 shows the effect of Zr-POSS on the curing characteristics of SR. When cured at 155 °C, the T_{90} of SR were 481 s. When fabricated with Zr-POSS, although the T_{90} of SR/Zr-POSS nanocomposites was slightly delayed, the effect of Zr-POSS on the curing for SR was negligible.

S2. Transmittance of SR/Zr-POSS nanocomposites

Transmittance is one of the most important factors for SR in the electronics and aerospace applications. Figure S1 showed the UV-Vis spectra of SR/Zr-POSS nanocomposites. The transmittance of SR near infrared region was markedly higher than that of UV region, and the transmittance located in 450 nm was 39.5%. When 1.0 phr Zr-POSS was added, the transmittance of SR/Zr-POSS-1 nanocomposites at 450 nm was 36.1%, which only fell by 3.4% compared to the blank SR. With the increase of Zr-POSS, the transmittance of SR/Zr-POSS nanocomposites decreased gradually, but the decline was modest. This illustrated that Zr-POSS had little effect on the transmittance of SR, which was of great significance for the preparation of light transparent coating or LED encapsulation adhesive.

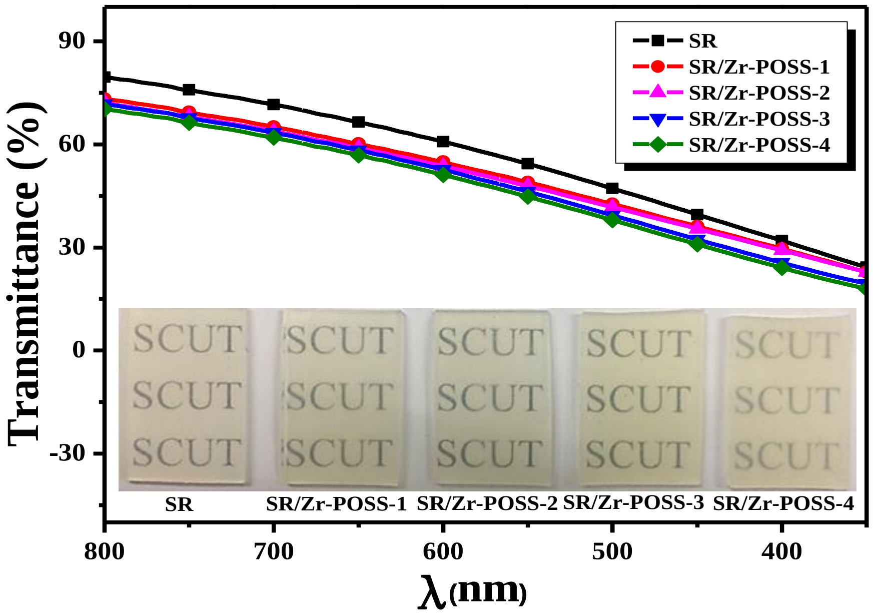

Figure S1. UV-Vis spectra of SR/Zr-POSS nanocomposites.

S3. Thermal stability of SR/Zr-POSS nanocomposites

Figure S2 presents the TGA and DTG curves of SR/Zr-POSS nanocomposites under nitrogen atmosphere, and the detailed results are listed in Table S2. SR began to decompose at 460.6 °C (T_5, temperature for 5% weight loss), which was mainly attributed to the homolytic cleavage of $Si\text{-}CH_3$.

With the increase of temperature, SR decomposed rapidly and reached a peak degradation at 657.0 °C (T_{max}), which were ascribed to the unzipping reaction of Si-OH end-group and the random scission reaction of backbone to release cyclic oligomer. When combined with 4.0 phr Zr-POSS, the T_5 of SR/Zr-POSS-4 nanocomposites were reached to 475.2 °C, and the T_{max} was increased from to 674.0 °C, meaning that Zr-POSS could remarkably improve the thermal oxidative stability of SR.

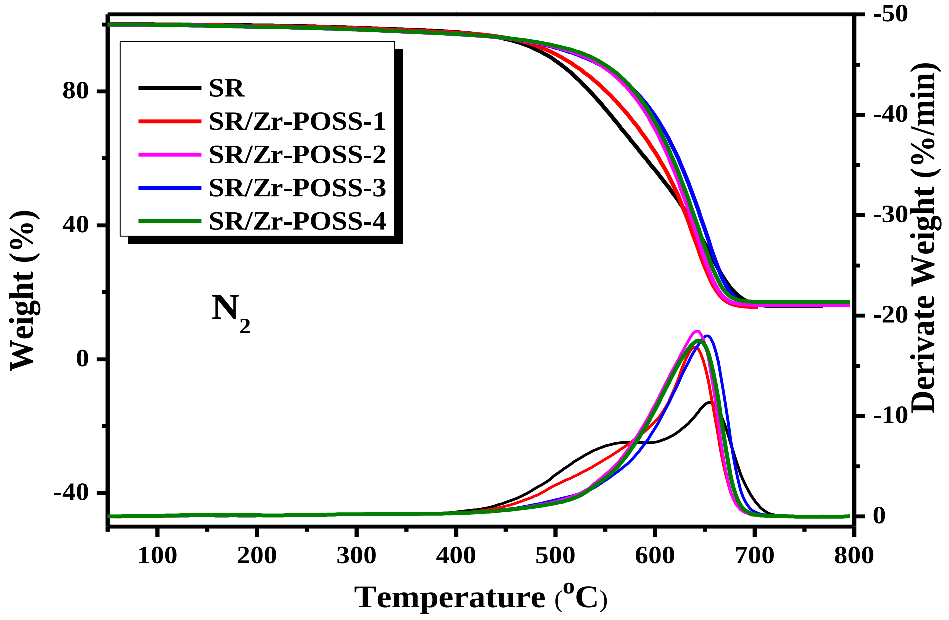

Figure S2. TGA and DTG curves of SR/Zr-POSS nanocomposites under nitrogen atmosphere.

Table S2. Characteristic data obtained from TGA and DTG curves (nitrogen, 20 °C/min).

**Samples.**	T_5 (°C)	T_{10} (°C)	T_{max} (°C)	**Residue at 700 °C (wt %)**
SR	460.6	498.0	657.0	15.8
SR/Zr-POSS-1	465.9	506.9	639.8	15.6
SR/Zr-POSS-2	470.9	530.1	652.2	16.2
SR/Zr-POSS-3	471.8	532.1	642.2	16.1
SR/Zr-POSS-4	475.2	537.2	674.0	17.0

For further investigation into the effect of Zr-POSS on the thermal degradation of SR, TG-FTIR was used to analyze the evolution of the pyrolysis products during the thermal degradation under nitrogen atmosphere. Figure S3 shows the 3D TG-FTIR and FTIR spectra of pyrolysis products for SR (a) and SR/Zr-POSS-4 nanocomposites (b) under nitrogen atmosphere. As could be seen from Figure S3, the cyclic oligomers (849 cm^{-1} and 1026 cm^{-1}) and methane (3017 cm^{-1} and 1304 cm^{-1}) were the main products for blank SR and SR/Zr-POSS-4 nanocomposites. The FTIR absorbance evolution of the two products decomposed by SR and SR/Zr-POSS-4 nanocomposites were presented in Figure S4. When 4.0 phr Zr-POSS was added, the degradation pathway of SR/Zr-POSS-4 nanocomposites showed some difference with SR. As showed in Figure S4(a), the release of methane was effectively suppressed by the addition of Zr-POSS. This could be explained by that the Zr and phenyl of Zr-POSS could capture the methyl radical produced by the homolysis of Si-CH_3, thus inhibit the generation of methane. In addition, the double release peaks of cyclic oligomers for SR became a single peak in SR/Zr-POSS-4 nanocomposites. This was due to the complexation between Zr and Si-OH, which could restrain the unzipping reaction of Si-OH end-group and protect the matrix from degradation.

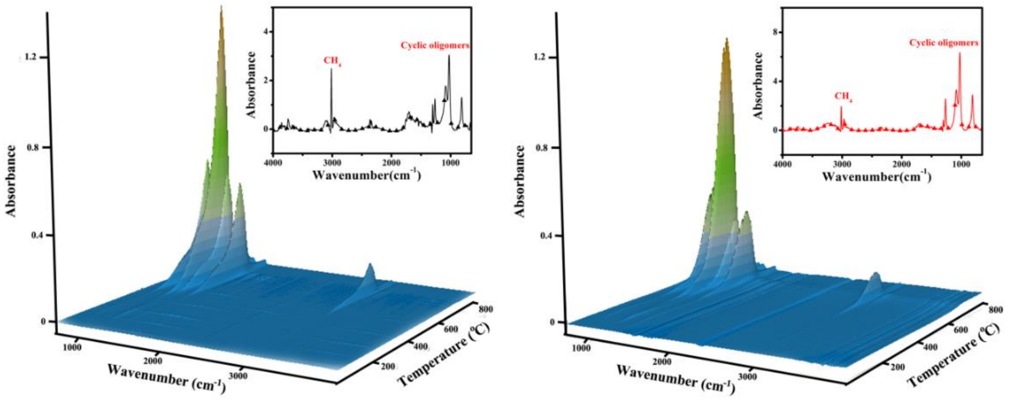

Figure S3 3D TG-FTIR and FTIR spectra of pyrolysis products for SR (**a**) and SR/Zr-POSS-4 nanocomposites (**b**) under nitrogen atmosphere.

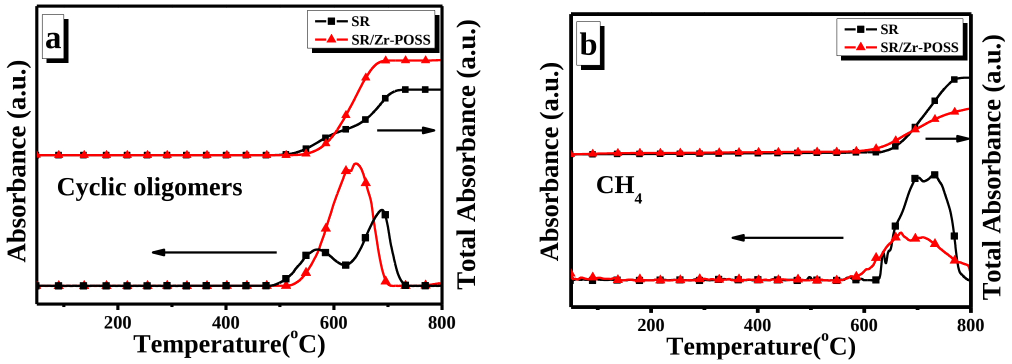

Figure S4 FTIR absorbance evolution of the pyrolysis products during thermal degradation for SR and SR/Zr-POSS-4 nanocomposites under nitrogen atmosphere.

S4. $^{2.9}$Si NMR

^{29}Si NMR spectra of SR and SR/Zr-POSS-4 were also used to analyze the effect of Zr-POSS on the thermal-oxidative aging of SR. The results are shown in Figure S5. The blank SR and SR/Zr-POSS-4 before aging were mainly characterized by the signals at −23.3 ppm, which belonged to the difunctional (D) Si structure. After aging, the side methyl group of blank SR underwent oxidative degradation and further crosslinking. This phenomenon was manifested as some new bands appeared at −57.8 and −67.9 ppm in Figure S5 (a), which were ascribed to the trifunctional (T) Si structure. However, when Zr-POSS was incorporated, the ^{29}Si NMR spectra of SR/Zr-POSS-4 were almost unchanged before and after aging, indicating that Zr-POSS could significantly suppressed the crosslink reaction of SR. This could be due to the thermal stability improvement of POSS and the effective capture of free-radical by the Zr of Zr-POSS.

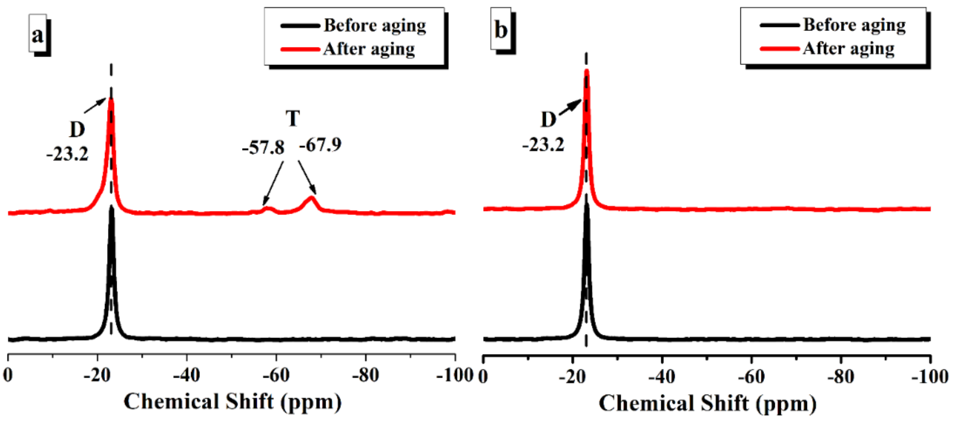

Figure S5. ^{29}Si NMR spectra of SR (**a**) and SR/Zr-POSS-4 nanocomposites (**b**) before and after aging.
